# Evaluation and management of worsening virilization in a postmenopausal woman diagnosed with PCOS

**DOI:** 10.1210/jendso/bvag110

**Published:** 2026-05-09

**Authors:** Matthew F Osborne, William B Horton, Arthur J Pesch, Richard J Santen

**Affiliations:** Division of Endocrinology and Metabolism, University of Virginia School of Medicine, Charlottesville, VA 22903, USA; Division of Endocrinology and Metabolism, University of Virginia School of Medicine, Charlottesville, VA 22903, USA; Department of Radiology and Medical Imaging, University of Virginia School of Medicine, Charlottesville, VA 22903, USA; Division of Endocrinology and Metabolism, University of Virginia School of Medicine, Charlottesville, VA 22903, USA

**Keywords:** testosterone, ovary, male pattern baldness, hormones

## Abstract

The manifestations of common disorders can infrequently obscure the presence of very rare conditions that share similar clinical findings. Polycystic ovary syndrome (PCOS) is a common disorder characterized by hyperandrogenism, ovulatory dysfunction, and/or polycystic ovarian morphologic features. A key component of the Rotterdam PCOS diagnostic criteria is the exclusion of other conditions that can mimic the signs and symptoms of PCOS. Compared to PCOS, androgen-producing tumors are rare neoplasms that originate in either the ovaries or adrenals and appear in only about 0.2% of people with hyperandrogenism. Ovarian steroid cell tumors (OSCTs) are an exceptionally rare subset of this class that represents less than 0.1% of all ovarian neoplasms. OSCTs are often hormonally active and classically cause the sudden and severe onset of secondary virilizing characteristics. In the case presented here, we describe a postmenopausal patient who was referred for management of PCOS but ultimately found to have an OSCT. A detailed history elicited a worsening of symptoms after menopause that raised the suspicion of a virilizing tumor; however, the clinical challenge was to determine whether the patient had both PCOS and an OSCT or whether the OSCT had been “causing” PCOS all along. This required careful scrutiny of the patient's 24-year clinical course and a comprehensive literature review in an effort to distinguish the manifestations of an OSCT from those of PCOS.

The manifestations of common disorders can infrequently obscure the presence of very rare conditions that share similar clinical findings. Polycystic ovary syndrome (PCOS) is a common disorder characterized by hyperandrogenism, ovulatory dysfunction, and/or polycystic ovarian morphologic features [1] that affects nearly 6% to 10% of reproductive-age women [1, 2]. A key component of the Rotterdam PCOS diagnostic criteria is the exclusion of other conditions (eg, congenital adrenal hyperplasia, androgen-secreting tumors) that can mimic the signs and symptoms of PCOS [2, 3]. Compared to PCOS, androgen-producing tumors are rare neoplasms that originate in either the ovaries or adrenals and appear in only about 0.2% of people with hyperandrogenism [4]. Ovarian steroid cell tumors (OSCTs) are an exceptionally rare subset of this class that represents less than 0.1% of all ovarian neoplasms [5]. OSCTs are often hormonally active and classically cause the sudden and severe onset of secondary virilizing characteristics [6].

In this Expert Endocrine Consult, we present a postmenopausal patient who was referred to us for management of PCOS but ultimately found to have an OSCT. A detailed history elicited a worsening of symptoms after menopause that raised the suspicion of a virilizing tumor; however, the clinical challenge was to determine whether the patient had both PCOS and an OSCT or whether the OSCT had been “causing” PCOS all along. This required careful scrutiny of the patient's 24-year clinical course and a comprehensive literature review in an attempt to distinguish the manifestations of OSCT from those of PCOS.

## Case presentation

A 62-year-old nulliparous postmenopausal woman was referred to our endocrinology clinic for evaluation of acute worsening of hirsutism in the presence of a 24-year history of PCOS. She reported menarche at age 11 years followed by long periods of oligomenorrhea and amenorrhea as well as hirsutism and diabetes. At age 60 years, she began experiencing irregular bleeding for 3 to 5 days every month requiring a dilation and curettage, which revealed hyperplastic endometrium. During our initial visit, the patient reported increased hair growth overall and new hair growth specifically on the chest, back, and legs over the preceding 2 to 3 months. She also noted that she previously shaved her face once weekly but now required daily shaving to limit hair growth. She reported lifelong obesity but denied any recent weight gain. She denied any use of androgenic medications. Other pertinent medical history included ductal carcinoma in situ of the breast (status post treatment with tamoxifen), papillary thyroid microcarcinoma (status post total thyroidectomy and now on levothyroxine for thyroid hormone replacement), hypertension, type 2 diabetes mellitus, hyperlipidemia, chronic obstructive pulmonary disease, and metabolic dysfunction–associated steatotic liver disease.

**Figure 1:**
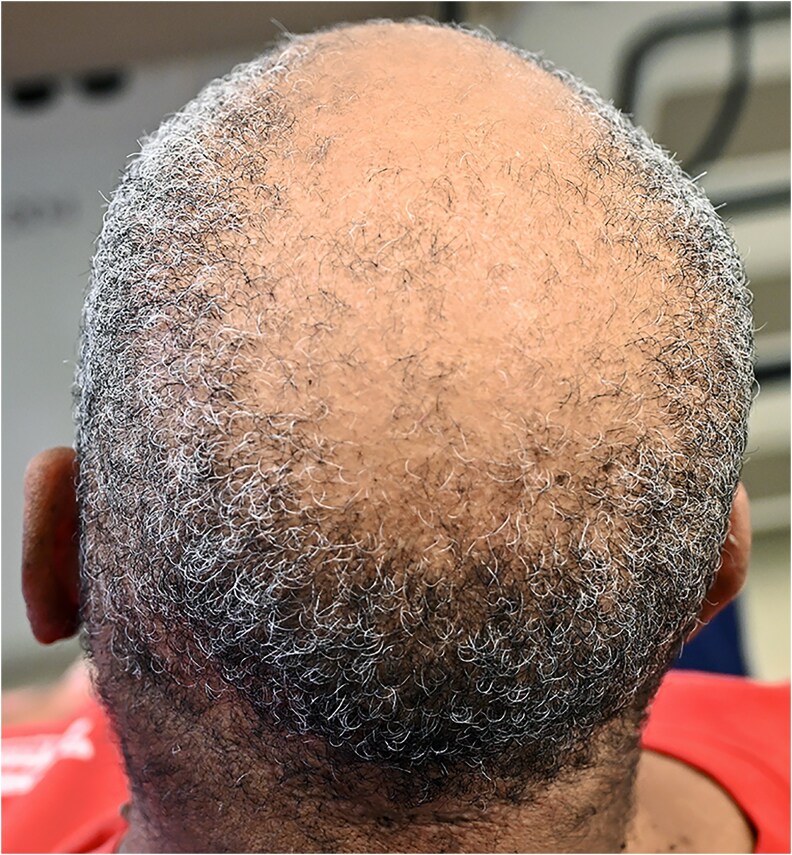
Ludwig stage 3 androgenic alopecia of the scalp.

**Figure 2:**
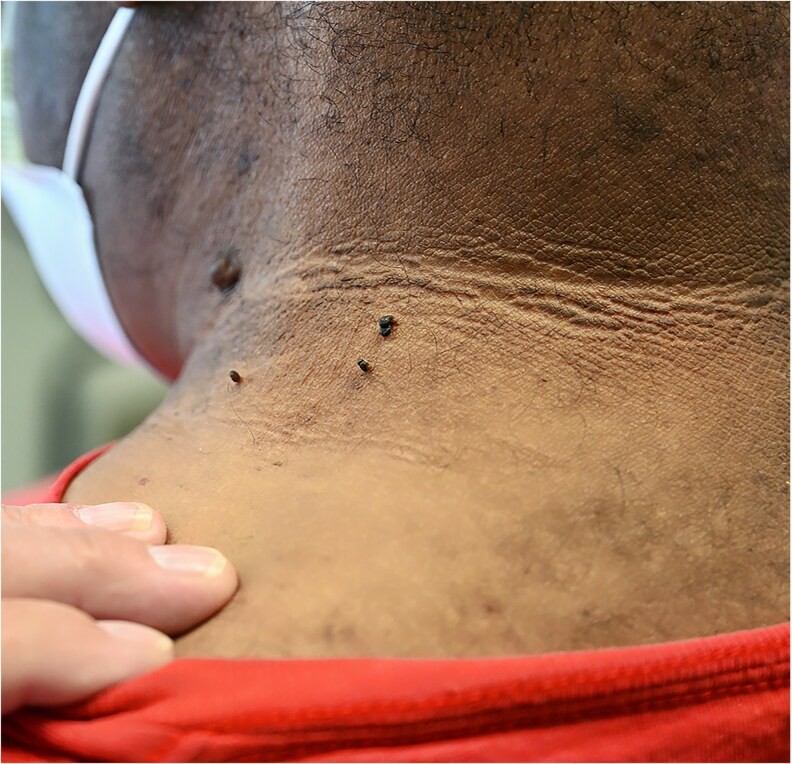
Acanthosis nigricans and skin tags noted along the posterior neck.

Initial physical examination was notable for hirsutism on the chin and upper lip, Ludwig stage 3 androgenic alopecia of the scalp (Fig. 1), acanthosis nigricans of the neck along with numerous skin tags (Fig. 2), and excess terminal hair growth on the chest, abdomen, back, legs, and arms. Genitourinary exam was deferred as gynecology had performed this exam 7 months prior and reported “prominent clitoromegaly.” Notably, clitoral enlargement had also been previously documented during a gynecology examination approximately 15 years prior to our initial visit with the patient. Examination for signs of Cushing syndrome revealed no proximal muscle weakness, dorsocervical fullness, or facial plethora. Vital signs were notable for hypertension (blood pressure of 130/83 mm Hg) and obesity (body weight of 212 pounds [∼96 kg] with body mass index of 36.1).

Our care team discussed the differential diagnosis and noted that PCOS was certainly a consideration given the patient’s clinical history (eg, oligomenorrhea, hirsutism) and physical examination results. However, the recent worsening of hirsutism, androgenic alopecia, and presence of clitoral enlargement raised the possibility of ovarian hyperthecosis along with ovarian and/or adrenal tumors [7, 8]. A key consideration was that patients with PCOS typically experience a decline in serum testosterone levels with age and regular ovulatory menses often resume as the patient approaches menopause [9-11]. With this in mind, we thought PCOS was an inadequate explanation for our patient's current clinical condition. Given that ovarian hyperthecosis is often associated with insulin resistance, diabetes, and metabolic syndrome, we favored this over the presence of a hilar cell adenoma [7, 12]. With the diagnosis still uncertain, we elected for further evaluation with hormonal biomarkers and dedicated imaging.

**Figure 3:**
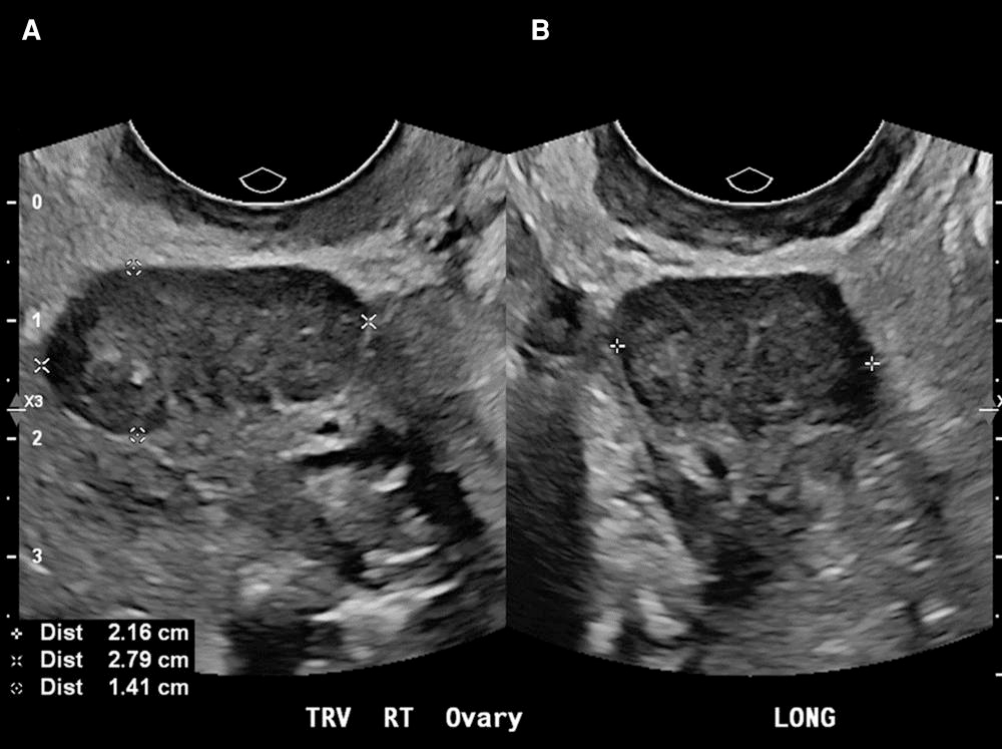
Grayscale transvaginal ultrasound (A, transverse view; B: longitudinal view) demonstrates a hypoechoic mass, measuring 2.2 × 2.8 × 1.4 cm, arising from the right ovary.

Biochemical evaluation revealed elevated total and free testosterone, undetectable estradiol, elevated luteinizing hormone and follicle-stimulating hormone (though these values were slightly lower than expected for typical postmenopausal female values, likely due to reintroduction of negative feedback on the hypothalamic-pituitary axis due to the elevated androgens), and slightly elevated androstenedione (Table 1). Magnetic resonance imaging of the ovaries demonstrated bilaterally enlarged ovaries for age with follicles and signal characteristics interpreted as compatible with benign stromal tumors including fibroma or Brenner tumors. Magnetic resonance imaging of the abdomen showed no evidence of adrenal tumors but did identify diffuse hepatic steatosis. A transvaginal pelvic ultrasound revealed the right ovary to be 2.1 × 2.9 × 2.5 cm with a hypoechoic 1.4 × 2.8 × 2.2 cm mass with scattered echogenicity (Fig. 3). The left ovary measured 2.7 × 1.7 × 2.7 cm, and while not well visualized, manifested a similar appearance of a hypoechoic mass (see Fig. 3).

**Table 1:** Biochemical assessment throughout evaluation and treatment

Encounter (y)	Total testosterone, ng/dL	Free testosterone, pg/mL	Estradiol, pg/mL	DHEAS, µg/dL	17-Hydroxyprogesterone, ng/dL	Androstenedione, ng/dL	LH, mIU/mL	FSH, mIU/mL	24-h urinary free cortisol, mcg/24 h
Initial PCOS diagnosis (April 2003)	105 (H)	—	—	—	—	—	—	—	
Visit to establish endocrine care in our system (July 2016)	89 (H)	15.2 (H)	—	70	<40	—	—	6.8	6.9
Initial referral visit for worsening hirsutism (April 2024)	103 (H)	26.4 (H)	<24	72.9	47	201 (H)	13.56	10.12	
During leuprolide acetate therapy (October 2024)	36	8.8 (H)	—	—		138	—	—	
Postbilateral oophorectomy (August 2025)	25	5.2	—	77.9		—	—	—	

Reference ranges reflect typical postmenopausal female laboratory values as reported by the performing laboratory. Elevated laboratory values are denoted by (H).

Abbreviations: DHEAS, dehydroepiandrosterone sulfate; FSH, follicle-stimulating hormone; GnRH, gonadotropin-releasing hormone; LH, luteinizing hormone; PCOS, polycystic ovary syndrome.

**Figure 4:**
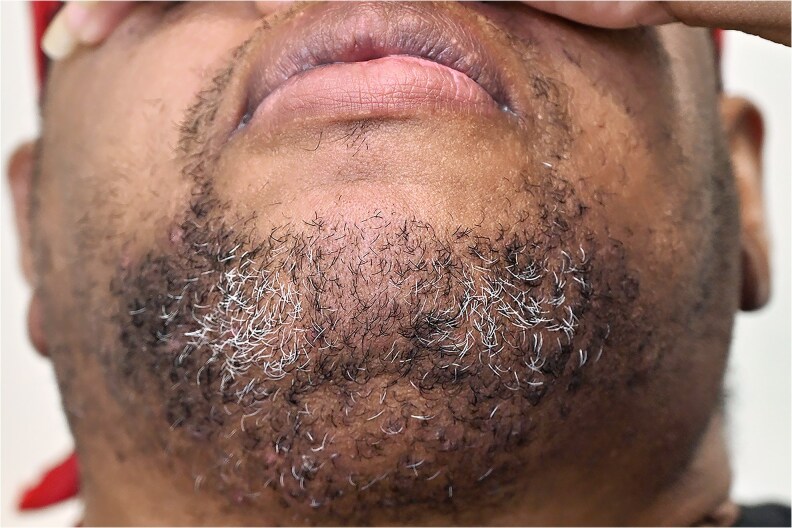
Facial hair growth noted after only 2 weeks without shaving.

**Figure 5:**
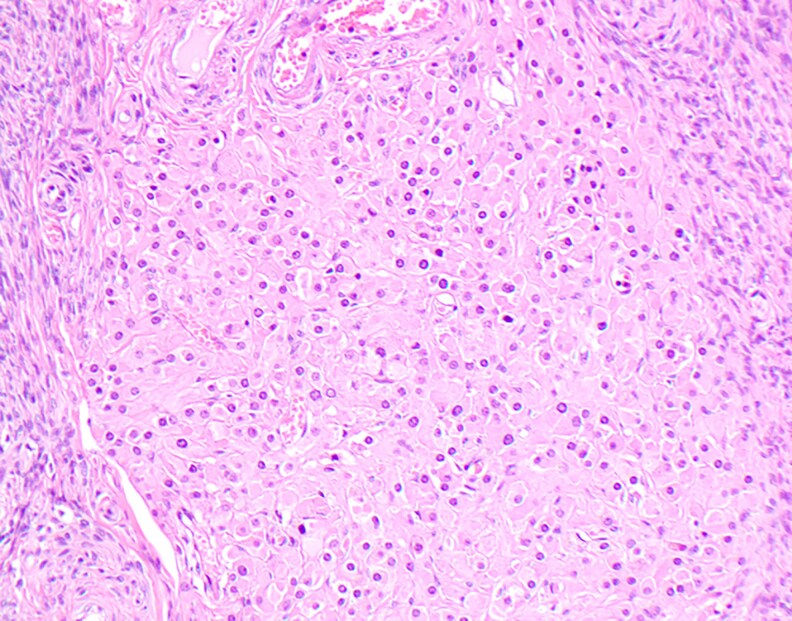
Pathological evaluation of the small right ovarian steroid cell tumor demonstrated polygonal to rounded tumor cells with distinct cell borders, central nuclei, and moderate to abundant eosinophilic cytoplasm.

Based on these results, we suspected that her hyperandrogenemia originated from the ovaries and administered leuprolide acetate for further evaluation. Biochemical results 1 month later showed markedly reduced total and free testosterone levels (see Table 1), supporting an ovarian source. We then asked her to return to clinic 2 weeks later without shaving her face and observed considerable hirsutism of the chin (Fig. 4). Bilateral salpingo-oophorectomy and hysterectomy were recommended as definitive therapy but delayed due to poorly controlled diabetes mellitus. While working to improve glycemic control, we continued leuprolide acetate injections and testosterone values continued to decrease (see Table 1). The patient’s glycemic control subsequently improved with intensified antidiabetic medical therapy and 1 year later she underwent robot-assisted total laparoscopic hysterectomy with bilateral salpingo-oophorectomy. Final surgical pathology revealed a small benign steroid-producing tumor in the right ovary (Fig. 5) and no abnormalities on the left. No malignant features were identified in either ovary. We then asked a pathologist with special interest in reproductive disorders to review our patient's pathology slides for hyperthecosis and no evidence of this was identified. Given the link between OSCTs and various genetic tumor syndromes [6, 13], we reviewed the chart and noted that the patient had undergone genetic testing prior to our visit. Sequence analysis and duplication/deletion testing was negative for any variants associated with pertinent genetic syndromes (including *APC*, *BRCA 1*, *BRCA 2*, *FH*, *VHL*, *RET*), with results confirmed by a consulting geneticist.

At follow-up approximately 6 months after surgical intervention, serum total testosterone and free testosterone had normalized. The patient reported mild regrowth of scalp hair in previously alopecic areas but hirsutism had only minimally improved. At her next follow-up visit 1 year later, there was no further improvement in alopecia or hirsutism (possibly due to chronic hair follicle changes and ongoing PCOS-related androgen excess that prevented complete resolution).

## Discussion

Herein we presented a challenging clinical case that highlights several valuable lessons for learners, trainees, and established clinical providers. First, a thorough history was vital to ascertain the fact that this patient's clinical signs and symptoms had acutely worsened in the postmenopausal state, which is atypical of PCOS. Failure to recognize this fact could have led to biases (eg, anchoring and confirmation) that may have prevented us from reaching an appropriate diagnosis and therapy. Thus, we emphasize the value of challenging diagnoses (both of oneself and others) when the clinical signs and symptoms do not fit neatly into the specific illness script. In similar fashion, the clinical conundrum in this case was to determine whether Sutton's law [14] applied. Sutton's law advises that when diagnosing, one should first consider the obvious. In our patient, that meant considering whether her initial PCOS diagnosis explained her constellation of signs and symptoms, whether she had PCOS and then developed an OSCT in the postmenopausal years, or whether her initial PCOS diagnosis had been incorrect and her prolonged history was entirely due to an OSCT. Answering this question required a detailed review of the medical literature.

The term *steroid-secreting tumor* was first coined in 1979 by Scully et al and included 3 subtypes [1]: stromal luteomas (∼30%) [2]; Leydig cell tumors involving both hilus and nonhilus cell subtypes (∼20%), and [3] not otherwise specified (∼50%) [15, 16]. These tumors are usually benign but can be malignant [6], with one retrospective clinicopathological case series reporting that 25% to 43% were clinically malignant [15]. The presence of more than 2 mitotic figures per 10 high-power fields suggests malignant potential [6, 17]. In postmenopausal women, the mean time to diagnosis is 5 years [6, 12] but some OSCTs have been reported to be present for as many as 30 years prior to diagnosis [18]. Between 5% and 6% are clinically associated with Cushing syndrome and the aromatization of androgens to estrogens can result in postmenopausal bleeding [6]. Macroscopically, the OSCTs are often yellow due to lipidic content and microscopically they have eosinophilic cytoplasm with nests of large rounded-to-polygonadal cells and round nuclei with prominent nucleoli [15, 16]. As noted earlier in our case presentation, histologic examination provides an opportunity to distinguish OSCTs from ovarian hyperthecosis. In contrast to OSCTs, hyperthecosis is usually characterized by the scattered presence of nests of luteinized theca cells in the ovarian stroma due to differentiation of the ovarian interstitial cells into steroidogenic luteinized stromal cells [19]. The ovaries are clearly enlarged up to 10 cm^3^ in comparison to normal postmenopausal ovaries, which are typically 1 to 5 cm^3^ [7].

Regarding clinical overlap between OSCTs and PCOS, virilization, weight gain, and amenorrhea along with elevated testosterone levels are common findings in both conditions [18]. Less common manifestations include hypertension, abdominal pain, vaginal bleeding, Cushing syndrome, and acanthosis nigricans [4, 5, 7, 12, 15, 16, 20-25]. While the various OSCT case series that we reviewed did not specifically comment on whether diabetes, acanthosis nigricans, skin tags, and/or metabolic syndrome are common features, several reports indicate that excess androgen production itself can induce these signs [7, 26]. At face value, these considerations suggest that the OSCT was the primary cause of our patient's clinical condition. However, the patient's long-standing metabolic disease with dermatologic manifestations make it unlikely that an OSCT was entirely responsible for her complex medical history.

We next carefully considered whether the patient met the diagnostic criteria for PCOS. The Rotterdam criteria require satisfaction of at least 2 of 3 components to make the diagnosis of PCOS [3]. Importantly though, the Rotterdam criteria also require exclusion of other conditions that mimic PCOS (such as ovarian tumors). When our patient received her initial PCOS diagnosis, she met the Rotterdam criteria [3], the National Institutes of Health criteria, and the Androgen Excess-PCOS Society criteria [27]. However, she also had clitoral enlargement on physical examination at that time and a key point here is that this sign is not typically associated with PCOS [7]. Others have emphasized the need to recognize that, in cases of PCOS wherein hyperandrogenic symptoms increase and/or develop into virilization, alternative causes of androgen excess must be ruled out [7]. In our view, this should have led to consideration of alternative diagnoses in the present case.

With the recognition that our patient had some signs that would be atypical for PCOS, we needed to determine whether she had an ovarian or adrenal source of hyperandrogenism to help refine our differential. Here we note that imaging studies of our patient demonstrated a small hypoechoic right ovarian mass. However, in cases where imaging is inconclusive, gonadotropin-releasing hormone suppression or selective venous (ie, ovarian and adrenal vein) sampling is recommended to help localize the source of androgen excess [28, 29]. One noted drawback of selective venous sampling, though, is that it requires technical expertise and has a high failure rate. Given the positive imaging in our patient, we eschewed venous sampling and thought that administration of leuprolide acetate would be instructive. Leuprolide acetate is a gonadotropin-releasing hormone agonist that can distinguish whether excess testosterone is originating from the ovaries or adrenals [30]. If serum testosterone is suppressed following administration, an ovarian source is confirmed given that such conditions are gonadotropin dependent [7, 30]. Conversely, if testosterone levels remain high or show minimal reduction despite suppressing ovarian function, the source is likely to be the adrenal glands [7, 30]. However, the test cannot differentiate between ovarian hyperthecosis and an OSCT given that both disorders are gonadotropin dependent and will respond to leuprolide acetate with testosterone inhibition [7, 31]. At this point, the patient's clinical history was again useful. While ovarian hyperthecosis is more common than OSCTs in postmenopausal women with hyperandrogenism (prevalence 9.3% vs 2.7%, respectively [32]), the two conditions have different clinical presentations. Here the patient's clinical history again proved highly informative. Ovarian hyperthecosis is characterized by gradual development of virilizing symptoms while OSCTs typically have a rapid onset of virilization [7, 32]. Our patient had a very short timeline for the worsening of her virilization symptoms, and recognizing this proved key to ultimately arriving at the proper diagnosis.

## Conclusion

In summary, we presented a challenging clinical case that highlights the value of thorough history taking and detailed knowledge of the clinical signs resulting from PCOS and/or OSCTs. As PCOS is a syndrome without a unique molecular marker allowing a specific diagnosis, we remain unsure whether PCOS and OSCT coexisted in this patient or whether her clinical condition was caused solely by the OSCT all along. Cases such as these challenge the diagnostic skills even of “expert” endocrinologists and highlight the value of thinking beyond an initial diagnosis so that appropriate evaluation and therapy can be pursued.

## Data Availability

Data sharing is not applicable to this article as no datasets were generated or analyzed during the case presentation.

## Abbreviations

OSCT: ovarian steroid cell tumor
PCOS: polycystic ovary syndrome
